# Fresh cassava root replacing cassava chip could enhance milk production of lactating dairy cows fed diets based on high sulfur-containing pellet

**DOI:** 10.1038/s41598-022-07825-w

**Published:** 2022-03-09

**Authors:** Rittikeard Prachumchai, Anusorn Cherdthong, Metha Wanapat, Sarong So, Sineenart Polyorach

**Affiliations:** 1 Tropical Feed Resources Research and Development Center (TROFREC), Khon Kaen University, Khon Kaen, 40002 Thailand; 2 Department of Animal Science, Faculty of Agriculture, Khon Kaen University, Khon Kaen, 40002 Thailand; 3 Department of Animal Science, Faculty of Agriculture and Food Processing, National University of Battambang, Battambang, 02352 Cambodia; 4 Department of Animal Production Technology and Fisheries, Faculty of Agricultural Technology, King Mongkut’s Institute of Technology Ladkrabang, Chalongkrung Road, Ladkrabang, Bangkok, 10520 Thailand

**Keywords:** Applied microbiology, Bacteria, Biotechnology, Animal physiology

## Abstract

The experiment objective was to assess the shifting effect from cassava chip (CC) to fresh cassava root (FC) affected feed utilization, rumen metabolism, cyanide-using bacteria, and milk quality in lactating Thai Friesian dairy cows fed diets based on high sulfur-containing pellet (PS). Four lactating Thai Friesian dairy cows of 481.5 ± 31.3 kg BW (about 4 years old were allocated with four treatments in a 4 × 4 Latin square design. The four treatments were: replacement FC for CC at 0%, 60%, 80%, and 100% dry matter (DM), respectively. Feed intakes for four diets in terms of total dry matter intake (kg/day and % BW) was linearly correlated with levels of replacement of FC (p < 0.01). Digestibilities of crude protein (CP), DM, organic matter (OM), amylase-treated neutral detergent fiber (aNDF), and acid detergent fiber (ADF) did not change with increasing levels of FC in the diet (p > 0.05). Moreover, the total bacterial counts and cyanide population utilizing bacteria cubically increased with an increase of FC replacement (p < 0.01). The effect of CC substitution with FC in the PS diet was cubically increased on blood thiocyanate concentrations (p < 0.01). In addition, the propionate (C3) concentration at 0 and 4 h post-feeding changed significantly among treatments (p < 0.01), which were linearly improved with an increasing dose of replacement FC and were highest when FC was replaced at 100%. The yield of 3.5% fat-corrected milk was high in the treatment with the replacement of FC as compared to the control (p < 0.01). The yield of fat and milk fat percentages was high (p < 0.01) in the group with the replacement of FC as compared to feed with no diet replaced. The milk thiocyanate concentration was cubically enhanced when levels of FC replacement increased (p < 0.01) and was the highest when FC was replaced at 100%. As the amount of FC replacement was raised, the somatic cell count in the milk decreased linearly (p < 0.01). In conclusion, the replacement of FC at 100% in PS could enhance the feed intake, microbial populations, total volatile fatty acid (VFA), C3 concentration, milk yield, and milk quality.

## Introduction

In Asian regions, including Thailand, cassava is commonly cultivated for human food and animal feed^1^. Cassava chip (CC) is a processed form of fresh cassava roots (FC) and a vital feed ingredient for ruminants; it is used approximately 40% to 60% in a formulation^2^. However, the high production of CC during the rainy season is inadequate to meet the demand. It has been claimed that the use of FC is appropriate in all seasons, available in an adequate amount, and obtainable for a low price^3^. However, cyanogenic compounds contained in FC, including linamarin and lotaustralin, are constraints for FC usage. Sulfur-dependent rhodanese has been found naturally in the rumen and can reduce cyanide toxicity by transforming into a less toxic substance in the form of thiocyanate^4,5^. It has been claimed that sulfur reduces cyanide toxicity, and it is recommended for a diet with low sulfur-containing ingredients (< 0.2% DM)^6^. The addition of sulfur to various forms of diets, including feed-block and total mixed ration (TMR), has been studied, and an increase in blood thiocyanate has been found^4^, although these methods of addition are complex, time-consuming, and costly. Pelleting is another method of feed processing and is easy to handle^1^.

Pelleting has been successfully used for ruminants due to its various advantages, including feed selection prevention, constituent-separating feed prevention, higher bulk density provision, and nutrient utilization improvement resulting in a better feed conversion ratio^2^. Pellet feeding has been shown to improve digestibility and modify ruminal fermentation patterns^7^. However, the effect of FC replaced CC in high sulfur-containing pellet (PS) on milk production, and its composition has not yet been evaluated.

Therefore, the study's objective was to evaluate the effect FC replacing CC on feed utilization, rumen metabolism, cyanide-using bacteria, and milk quality in lactating Thai Friesian dairy cows fed diets based PS. This study hypothesized that FC replacing CC could enhance feed efficiency, rumen characteristics, milk yield, and milk quality in cows fed diets based PS.

## Results

### Dietary detail

FC was used to replace CC as the main energy source and contained 0.3 g/kg DM of hydrogen cyanide (HCN). The concentrate was formulated to contain 16% of CP, and RS as roughage feed was provided.

### Intake and digestibility

The effects of replacing CC with FC on feed intake and total-tract apparent digestibility of lactating Thai Friesian dairy cows are listed in Table 1. Feed intakes for four diets in terms of total dry matter intake (kg/day and % BW) were linearly correlated with levels of replacement of FC (p < 0.01), and the higher values were in the group with replacement of FC for CC at 100%. Intakes of FC and HCN were cubically significantly increased based on the level of FC replacement (p < 0.05), while the sulfur intake was similar among FC replacement groups (p > 0.05). The total-tract apparent digestibility for all parameters and nutrient intake of OM, CP, amylase-treated neutral detergent fiber (aNDF), and acid detergent fiber (ADF) was not significantly different among treatments (p > 0.05), while an increased level of FC in PS linearly increased the nutrient intake of DM (p < 0.01).

**Table 1:** Nutrient intake and total-tract apparent digestibility in lactating Thai Friesian dairy cattle fed the replacement of cassava chip by fresh cassava root (FC).

Item	FC replacement (%)				SEM	Contrast		
	0	60	80	100		L	Q	C
**Total dry matter intake**								
kg/day	12.38^a^	13.07^b^	13.25^b^	13.81^c^	0.17	< 0.01	0.72	0.26
%BW	2.57^a^	2.71^b^	2.75^b^	2.87^c^	0.03	< 0.01	0.71	0.25
FC intake kg/day	0.00^a^	2.19^b^	2.96^c^	3.71^d^	0.10	< 0.01	< 0.01	< 0.01
Sulfur intake kg/day	0.178	0.186	0.184	0.196	0.006	0.08	0.71	0.44
HCN intake g/day	0.00^a^	0.863^b^	1.751^c^	2.279^d^	0.02	< 0.01	< 0.01	< 0.05
**Nutrient intake, kg/day**								
Organic matter	10.86	11.36	11.40	11.96	0.45	0.13	0.94	0.64
Crude protein	1.01	1.11	1.11	1.09	0.03	0.13	0.12	0.65
aNeutral detergent fiber	5.75	5.79	6.01	6.46	0.35	0.18	0.69	0.80
Acid detergent fiber	3.68	3.81	3.88	4.16	0.32	0.31	0.81	0.84
**Digestibility coefficients, %**								
Dry matter	66.90	67.20	68.01	68.74	0.71	0.07	0.77	0.85
Organic matter	69.77	70.16	71.60	71.68	0.85	0.08	0.86	0.53
Crude protein	65.73	67.30	67.65	66.86	2.45	0.74	0.64	0.99
aNeutral detergent fiber	56.37	59.30	58.86	58.92	1.48	0.30	0.35	0.57
Acid detergent fiber	44.03	48.29	47.05	48.63	1.62	0.11	0.43	0.27

^a,b,c,d^Means in the same row with different superscripts differ (p < 0.05). L = linear, Q = quadratic, C = cubic, FC = fresh cassava root, HCN = hydrogen cyanide, SEM = standard error of the mean.

### Ruminal ecology and microorganism

The ruminal fermentation and microorganism populations at 0 and 4 h post-feeding and the mean values are listed in Table 2. Ruminal pH was similar among treatments (p > 0.05), which showed a consistent range of 6.74 to 6.83 for overall means. Furthermore, the ammonia nitrogen (NH_3_-N) concentration did not increase with the level of FC replacement (p > 0.05). In this study, the protozoal population at 0 and 4 h post-feeding was not significantly different among treatments (p > 0.05). In contrast, the total bacterial counts and cyanide population utilizing bacteria at 0 and 4 h post-feeding and the mean values were cubically increased with the dose of FC replacement (p < 0.01), which was highest in the group with replacement of FC at 100%.

**Table 2:** Rumen ecology and microorganisms (log10/g digesta) in lactating Thai Friesian dairy cattle fed the replacement of cassava chip by fresh cassava root (FC).

Item	FC replacement (%)				SEM	Contrast		
	0	60	80	100		L	Q	C
**Rumen ecology**								
Ruminal pH								
0 h post feeding	6.90	6.89	6.89	6.85	0.02	0.17	0.49	0.63
4 h post feeding	6.74	6.76	6.60	6.63	0.06	0.13	0.93	0.24
Mean	6.82	6.83	6.74	6.74	0.07	0.12	0.91	0.46
Ammonia–nitrogen concentration, mg/dL								
0 h post feeding	15.87	14.47	13.34	13.11	0.94	0.07	0.56	0.88
4 h post feeding	18.11	17.24	16.92	16.65	1.01	0.34	0.78	0.91
Mean	16.99	15.85	15.13	14.88	0.99	0.06	0.54	0.98
**Ruminal microbes, cell/mL**								
Protozoa, log_10_								
0 h post feeding	5.16	4.79	4.94	5.01	0.36	0.87	0.57	0.73
4 h post feeding	5.20	4.91	4.98	4.94	0.38	0.69	0.75	0.80
Mean	5.18	4.85	4.96	4.98	0.38	0.68	0.53	0.67
Bacteria, log_10_								
0 h post feeding	9.52^a^	9.56^b^	9.57^c^	9.62^d^	0.002	< 0.01	0.46	< 0.01
4 h post feeding	9.54^a^	9.58^b^	9.58^b^	9.63^c^	0.002	< 0.01	< 0.05	< 0.01
Mean	9.53^a^	9.57^b^	9.58^b^	9.62^c^	0.003	< 0.01	0.23	< 0.01
Cyanide utilizing bacteria, log_10_								
0 h post feeding	4.45^a^	4.97^b^	5.08^c^	5.17^d^	0.01	< 0.01	< 0.01	< 0.01
4 h post feeding	4.78^a^	5.11^b^	5.19^c^	5.25^d^	0.01	< 0.01	< 0.01	< 0.01
Mean	4.61^a^	5.04^b^	5.13^c^	5.21^d^	0.03	< 0.01	< 0.01	< 0.01

^a,b,c,d^Means in the same row with different superscripts differ (p < 0.05). L = linear, Q = quadratic, C = cubic, FC = fresh cassava root, SEM = standard error of the mean.

### Blood metabolites and ruminal fermentation

Blood metabolites in the lactating Thai Friesian dairy cow fed different doses of FC as a replacement for CC are listed in Table 3. Blood urea nitrogen (BUN) concentration did not differ among the four treatments with different doses of FC replacement (p < 0.05). However, the effect of CC substitution with FC was cubically increased on blood thiocyanate concentrations at 0 and 4 h post-feeding (p < 0.01), what was highest when 100% of FC was replaced. The dose of FC replacement did not alter C2 concentrations at 0 and 4 h post-feeding and the mean value (p > 0.05). However, the total volatile fatty acid (VFA), C3, and C4 concentration changed significantly among treatments (p < 0.01), which were linearly correlated with an increasing dose of replacement FC at 100%.

**Table 3:** Blood metabolites and ruminal volatile fatty acid (VFA) profiles in lactating Thai Friesian dairy cattle fed the replacement of cassava chip by fresh cassava root (FC).

Item	FC replacement (%)				SEM	Contrast		
	0	60	80	100		L	Q	C
**Blood parameters**								
Blood urea-N concentration, mg/dL								
0 h post feeding	13.00	12.25	12.00	12.00	1.53	0.65	0.81	0.97
4 h post feeding	15.00	14.50	14.00	13.50	1.12	0.36	0.99	0.99
Mean	14.00	13.38	13.00	12.75	1.11	0.25	0.81	0.97
Blood thiocyanate concentration, mg/dL								
0 h post feeding	2.00^a^	14.38^b^	20.50^c^	29.88^d^	0.81	< 0.01	0.11	< 0.05
4 h post feeding	2.63^a^	20.75^b^	26.25^c^	35.25^d^	0.99	< 0.01	< 0.01	< 0.01
Mean	2.31^a^	17.56^b^	23.38^c^	32.56^d^	1.00	< 0.01	< 0.01	< 0.01
Total VFA, mmol/L								
0 h post feeding	101.19	102.39	104.71	105.63	1.90	0.12	0.94	0.78
4 h post feeding	104.24	106.03	107.08	107.79	1.60	0.15	0.74	0.96
Mean	102.71^a^	104.21^ab^	105.89^ab^	106.71^b^	1.54	< 0.05	0.76	0.83
**VFA profiles, mol/100 mol**								
Acetic acid								
0 h post feeding	66.76	66.22	65.30	65.32	1.17	0.36	0.82	0.81
4 h post feeding	64.90	64.37	63.63	63.29	0.51	0.06	0.86	0.80
Mean	65.83	65.30	64.47	64.31	0.75	0.06	0.73	0.69
Propionic acid								
0 h post feeding	23.64^a^	24.22^a^	25.63^b^	26.44^b^	0.34	< 0.01	0.75	0.38
4 h post feeding	24.63^a^	26.32^b^	26.22^b^	27.81^c^	0.30	< 0.01	0.87	0.06
Mean	24.13^a^	25.27^b^	25.93^c^	27.13^d^	0.31	< 0.01	0.88	0.31
Butyric acid								
0 h post feeding	9.60^ab^	10.56^a^	9.07^bc^	8.24^c^	0.36	< 0.05	< 0.05	0.09
4 h post feeding	10.47	9.56	10.15	8.90	0.83	0.31	0.84	0.40
Mean	10.04^a^	10.06^a^	9.61^ab^	8.57^b^	0.64	< 0.05	0.37	0.97

^a,b,c,d^Means in the same row with different superscripts differ (p < 0.05). L = linear, Q = quadratic, C = cubic, FC = fresh cassava root, SEM = standard error of the mean.

### Milk production and thiocyanate

The effect of CC substitution by FC on milk performance is listed in Table 4. Yield 3.5% fat-corrected milk was high in the treatment with the replacement of FC as compared to the control (p < 0.01), while the milk yield was similar among treatments with the inclusion of FC and CC, ranging from 13.06 to 13.82 kg/day. The yield of fat and milk fat percentages was high (p < 0.01) in the group with the replacement of FC as compared to the feed with no diet replaced. Meanwhile, the treatments did not affect the milk protein and milk lactose. The milk thiocyanate concentration was cubically enhanced when levels of FC replacement increased (p < 0.01) and was highest when FC was replaced at 100%. Moreover, increasing levels of FC replacement did not change MUN (p > 0.05). In contrast, the somatic cell counts in the milk were linearly decreased when the level of replacement FC increased (p < 0.01).

**Table 4:** Milk production and components in lactating Thai Friesian dairy cattle fed the replacement of cassava chip by fresh cassava root (FC).

Item	FC replacement (%)				SEM	Contrast		
	0	60	80	100		L	Q	C
**Yield, kg/day**								
Milk yield	13.06	13.17	13.75	13.82	0.49	0.21	0.96	0.66
3.5% FCM	12.15^a^	13.14^a^	13.93^b^	14.06^b^	0.55	< 0.05	0.45	0.86
Fat	0.41^a^	0.46^b^	0.49^b^	0.50^b^	0.02	< 0.05	0.33	0.95
Protein	0.50	0.49	0.53	0.52	0.03	0.45	0.93	0.49
Lactose	0.54	0.58	0.60	0.61	0.03	0.07	0.58	0.98
**Milk composition (%)**								
Fat	3.12^a^	3.49^a^	3.58^b^	3.60^b^	0.13	< 0.01	0.19	0.72
Protein	3.84	3.72	3.83	3.76	0.15	0.87	0.86	0.54
Lactose	4.15	4.39	4.36	4.40	0.11	0.19	0.39	0.52
Milk urea nitrogen, mg/dL	13.71	12.40	11.18	9.82	1.27	0.06	0.98	0.97
Milk thiocyanate, mg/dL	1.92^a^	10.48^b^	12.38^c^	14.58^d^	0.23	< 0.01	< 0.01	< 0.05
Somatic cell count, ×10^5^ cell/mL	5.71^a^	4.33^b^	3.11^c^	1.58^d^	0.35	< 0.01	0.84	0.78

^a,b,c,d^Means in the same row with different superscripts differ (p < 0.05). L = linear, Q = quadratic, C = cubic, FC = fresh cassava root, FCM = fat-corrected milk, SEM = standard error of the mean.

## Discussion

### Diet details

It was recommended that 0.2% dietary sulfur was required to maximize microbial growth and 0.4% was the tolerable level^8^. However, an additional 0.75% of sulfur showed no negative impact on animals and was effective to detoxify 1.46 g HCN/day^9^. In this study, HCN intake was 2.26 g/day, thus the addition of high sulfur in dairy cows’ diets was necessary to respond to the high intake of HCN.

In this study, FC contained 0.30 g/kg DM of HCN which was in the range of 0.24 to 0.33 g HCN/kg DM^8^. Dagaew et al.^3^ reported that FC contained 0.27 g/kg DM of HCN. The variation in HCN content caused variou factors such as climate change and geography^8^. Similarly, Supapong et al.^4^ found that FC contained 110 ppm of HCN; however, the chemical composition of the FC might vary depending on factors such as variety, soil fertility, state of growth, etc.

### Intake and digestibility

An increase in FC replacement linearly increased total DM intake. The physical characteristic (freshness, flavor, color, texture) difference between FC and CC may be the reason for the enhanced intake, suggesting that animals prefer FC to CC^1^. Moreover, feeding PS may enhance the intake regarding its physical form^10^. CC replaced with 100% FC showed no negative impact on intake. This could be explained by the sulfur addition, which functions as an HCN detoxifier. It has been reported that sulfur is vital for microbial growth (0.2% of dietary DM) and HCN detoxification which may require higher than 0.2% of dietary DM^8^. Therefore, 1.5% of sulfur was suitable for cows fed a diet containing HCN source ingredients^11^. Previous research into feeding FC containing high levels of HCN (160 ppm/h/day) with a feed block containing high sulfur to cattle demonstrated no negative impacts on feed intake^12^. Prachumchai et al.^13^ showed that daily HCN intake from 217.45 to 313.01 ppm showed no effect on intake. This could be due to the sulfur addition, which is a benefit for the detoxification of HCN by ruminal microbes^3,10^.

This study showed that digestibility did not change with the replacement of FC up to 100% compared with the control group; this could be because FC contains a high concentration of nonstructural carbohydrates similar to CC, quickly breaking down in rumen^3^. Supapong et al.^4^ indicated that replacing CC with FC for beef cattle fed on an TMR diet at 40% DM with 2% of sulfur maintains the digestibility of nutrients and rumen microbes. Similarly, Promkot and Wanapat^9^ revealed that 0.4% of sulfur did not impact intake and digestibility in cows fed a diet containing fresh cassava foliage. Cherdthong et al.^12^ showed that FC could be fed up to 1.5% BW with 1.5% sulfur supplementation. Thus, FC could replace CC up to 100% with PS feeding without a negative impact.

### Rumen ecology patterns and microorganism populations

Ruminal pH is one of the main factors determining the activity of microbes too-high or low-pH has a significant impact on microbe activity^4^. However, it was in the suitable range for microbial synthesis and activity to ferment the feed in the rumen^3^. Increasing FC replacement levels did not affect the NH_3_-N concentration. The optimal NH_3_-N concentration in the rumen ranged from 15 to 30 mg/dl^14^. Qi et al.^15^ revealed that FC feeding did not affect the concentration of NH_3_-N in goats. In steers, 1.5% FC resulted in no effect on NH_3_-N concentration with a high level of sulfur at 2% in feed blocks.

Increased FC replacement levels resulted in an increase in the bacterial population; this may be because of increased feed intake of DM, which increased the energy intake^3^. Supapong et al.^4^ reported that cattle fed 40% DM of FC resulted in an increase in the ruminal bacterial population. An increase in FC replacement up to 100% resulted in an increase in cyanide-utilizing bacteria compared to non-replacement of FC. This suggests that ruminal bacteria could use HCN for their growth^10^. Razanamahandry et al.^16^ reported that HCN acted as a nitrogen source for rhodanese and mercaptopyruvate sulfurtransferase. Kang and Kim^17^ showed that HCN concentration decreased in the medium with the increase in the bacterial growth rate.

### Blood metabolites and ruminal volatile fatty acid profiles

The average BUN concentration in dairy cows did not change when the animals were fed for four treatments in the current experiment. This suggested that BUN concentration is positively correlated with ruminal NH_3_-N^1^. Supapong and Cherdthong^5^ reported that the BUN concentration ranged from 10 to 15 mg/dl in cows fed TMR containing 40% DM of FC. BUN concentration is positively correlated with ruminal NH_3_-N^2^. In addition, overall mean blood thiocyanate increased by 92.90% with dairy cows fed on 100% replacement level of FC compared to the no-FC replaced group, which could be because high HCN consumption from FC resulted in conversion to the thiocyanate. Increasing FC replacement resulted in an increase in plasma thiocyanate, which suggested that rhodanese and sulfurtransferase converted HCN into thiocyanate, then were excreted via the urine^9^. NRC^8^ suggested that sulfur may require higher than the recommendation if the animal was fed a diet containing HCN ingredients.

Our results showed that the average propionate (C3) concentration increased by 11.06% when dairy cow fed on 100% replacement level of FC compared to the no-FC replaced group. Increasing C3 concentration could relate to the increase in intake and microbial population in the rumen. Similarly, Sutton et al.^18^ reported that increasing the feed intake of the DM resulted in higher rumen C3 concentrations. Wanapat and Khampa^2^ revealed that C3 concentration increased with FC supplementation as a soluble carbohydrate source in cows. In steers, 1.5% BW of FC increased C3 concentration with a feed block containing sulfur supplementation^12^.

### Milk performance, somatic cells count, and milk thiocyanate

Compared to the control, FC replacement significantly increased 3.5% fat-corrected milk and milk fat, which might occur because the difference in milk yield when the level of FC increased could be associated with the significant difference in feed intake and DM digestibility. In addition, higher performance could be explained by the higher C3 concentration absorbed upon entry into the liver via gluconeogenesis^10^. However, some previous studies did not show any changes in milk yield^5,9^. Milk fat concentration was affected upon the addition of FC with PS. Dageaw et al.^19^ reported that similar lactose, fat, protein, and casein production increased for cows receiving FC 1.5% BW as compared to the 1.0% BW group. Similarly, Supapong and Cherdthong^5^ observed that milk fat increased 4.0% when TMR containing 40% DM of FC was fed to the dairy cow. Our results showed that the milk thiocyanate concentration increased with the level of FC replacement. The higher levels of thiocyanate in the milk of cows feeding on FC in this study are attributed to a higher intake of HCN, which was then detoxified to thiocyanate (which is a metabolic result of HCN detoxification)^9^. In the current data, the milk thiocyanate concentration ranged from 1.92 to 14.58 mg/dl, which was around 2.2 times lower than blood thiocyanate. This finding might be due to the slow passage of thiocyanate from the bloodstream to the milk. According to Grun et al.^20^, the thiocyanate level in blood plasma was higher in lactating cows with stable udders than it was in milk (on average twice). Jose^21^ revealed that the mammary gland barrier reduces the passage of thiocyanate from maternal serum to milk.

Furthermore, the advantage of a significant dose of HCN in FC is that it changes the thiocyanate by eliminating feed HCN in the livers and kidneys of cows through the action of the rhodanese enzyme. Animals' livers and kidneys produce the rhodanese enzyme, which is involved in the conversion of HCN to thiocyanate in milk^19^. In a regression model between milk thiocyanate concentration and HCN in dietary FC findings, Supapong and Cherdthong^5^ stated that effects on milk thiocyanate concentration in dairy cows were also positively associated.

The reduction in somatic cell count could be due to the partition of blood thiocyanate into the milk which had antimicrobial property^5^. Meanwhile, the antimicrobial activity of the lactoperoxidase system, which is activated by the milk thiocyanate concentration, was affected to reduce the increase in the somatic cell count. Promkot and Wanapat^9^ previously demonstrated that the milk thiocyanate concentration and somatic cell count in the milk can be activated by lactoperoxidase as an indication of mastitis. Thus, by inhibiting microbial development, milk thiocyanate concentration from HCN can be used to extend the shelf life of raw milk processed at room temperature^19^. Similarly, Supapong and Cherdthong^5^ found that effects on somatic cell count in milk decreased 15.2% when TMR containing 40% DM of FC and 2% sulfur was fed to the dairy cow. Therefore, the results demonstrated that the replacement of FC at 100% could be beneficial in reducing somatic cell count and improving milk performance for the dairy cow.

It is possible to infer from this research that the replacement of CC with FC can manipulate rumen fermentation patterns. The replacement of FC at 100% could enhance the feed intake, total VFA, C3 concentration, microbial populations, and rate of disappearance of HCN in dairy cows fed with PS. Furthermore, milk yield and milk fat were increased, whereas somatic cell count was reduced when the replacement level of FC was increased. Further research should elucidate the effect of replacing CC with FC in PS on production trials with a high number of cows and a longer period than in the present study.

## Methods

Ethical-ACUC-KKU 11/2563 was approved by the Committees of Animal Care and Ethic of Khon Kaen University, based on the Ethics of Animal Experimentation of the National Research Council of Thailand. Our study confirmed that all methods were performed in accordance with the relevant guidelines and regulations.

### High sulfur-containing pellet preparation

Pellet ingredients were provided in Table 5. They were milled (Cyclotech Mill, Tecator, Hoganas, Sweden) into 0.1-mm. After milling, 1.5% DM of elemental sulfur powder was added and mixed roughly. Prior to pelleting, the mixed ingredient was adjusted to 20 to 25% of moisture. Finally, the PS was adjusted to 8 to 10% of moisture under the sun prior to feeding^7^.

**Table 5:** Ingredient and chemical composition of experimental diets, high sulfur-containing pellet (PS), fresh cassava root (FC), cassava chips (CC), and rice straw (RS) in the experiment.

Ingredients	FC replacement (%)				PS	FC	CC	Rice straw
	0	60	80	100				
Cassava chips	54.7	32.8	10.9	0				
Fresh cassava root	0.0	21.9	43.8	54.7				
**High sulfur-containing pellet**								
Soybean meal	19.0	19.0	19.0	19.0				
Rice bran	12.0	12.0	12.0	12.0				
Coconut meal	4.0	4.0	4.0	4.0				
Palm kernel meal	4.0	4.0	4.0	4.0				
Minerals and vitamins	1.0	1.0	1.0	1.0				
Sulfur powder	1.5	1.5	1.5	1.5				
Urea	1.5	1.5	1.5	1.5				
Salt	1.0	1.0	1.0	1.0				
Molasses	1.3	1.3	1.3	1.3				
**Chemical composition**								
Dry matter, %	91.1	90.8	90.4	90.1	91.4	35.1	94.2	92.9
Organic matter, %DM	92.2	91.5	91.9	92.5	85.5	96.8	96.3	89.6
Crude protein, %DM	16.0	16.1	16.2	16.0	32.3	2.3	2.3	1.7
aNeutral detergent fiber, %DM	20.1	19.5	19.1	18.8	25.2	11.2	11.6	72.5
Acid detergent fiber, %DM	12.6	12.3	12.0	11.9	17.8	6.1	6.3	46.7
Sulfur, %DM	1.44	1.42	1.39	1.42	32.5	ND	ND	ND
HCN, g/kg DM	ND	ND	ND	ND	ND	0.30	ND	ND

FC = fresh cassava root, DM = dry matter, HCN = hydrogen cyanide, ND = not determined. Minerals and vitamins (per kg): Vitamin A = 10,000,000 IU, Vitamin E = 70,000 IU, Vitamin D = 1,600,000 IU, Fe = 50 g, Zn = 40 g, Mn = 40 g, Co = 0.1 g, Cu = 10 g, Se = 0.1 g, I: 0.5 g.

### Fresh cassava root

FC, Manihot esculenta Kasetsart 50, was obtained from a supplier in Khon Kaen province. It was sliced to 0.2 to 0.4 mm (KR Strength Co., Ltd., Nakhon Ratchasima, Thailand) and fed at its respective amount after the pellet was fed.

### Cows, design, and feeding

Four Thai Friesian dairy cows of 481.5 ± 31.3 kg BW, and pre-experiment milk yield was 12.91 ± 0.52 kg/day, during the mid-lactation period were used and individually housed in 5 × 5 m pens equipped with clean water and mineral blocks (Fe = 50 g/kg, Zn = 40 g/kg, Mn = 40 g/kg, Co = 0.1 g/kg, Cu = 10 g/kg, Se = 0.1 g/kg, I: 0.5 g/kg). Each of the animals were randomly assigned to receive sulfur doses at 0.75% in diet. This study was designed using to a 4 × 4 Latin square design, consisting of four periods and treatments. Each period lasted for 21 days, of which 14 days were for an adaptation of treatments and 7 days were for the data collection. The treatments were the levels of FC replaced for CC at 0%, 60%, 80%, and 100% dry matter (DM), respectively. Cows were fed following the nutrient requirement recommended by NRC^8^. The concentrate (CC, FC, and PS) was fed to the cows at a 2:1 ratio (2 kg concentrate per 1 kg of milk yield) and rice straw was provided ad libitum. Diets were fed twice daily: at 06:30 a.m. and 3:30 p.m. Cows were weighed periodically to adjust the DM intake. Dietary intake and milk yield were recorded daily.

### Sampling and analysis

Feed and refusal samples were collected for 7 days of each period by compositing by cows and periods and freezing until use. The spot-sampling method was used for fecal sample collection. Fecal samples were composited by cows and periods and frozen until analysis. Feed, refusal, and fecal samples were defrosted under ambient temperature and dried in a hot-air oven for 72 h to analyze the nutrient content. After drying, the samples were ground into a powder form through a 1-mm screen (Wiley mill, PDS Chiangmai Engineering Co., Ltd., Chiangmai, Thailand) and analyzed for ash (ID942.05), DM (ID 967.03), and crude protein (CP, ID984.13)^22^. In addition, fiber components including aNDF and ADF were analyzed according to Cherdthong et al.^12^. To estimate the coefficient digestibility of nutrients, acid-insoluble ash content (AIA) was analyzed in the rations and fecal samples and used 4 N HCl as described the calculation following the proposed formulation of Van Keulen and Young^23^. The total sulfur content in the samples was determined by wet digestion with HNO_3_ and HClO_4_ (Sanford and Lancaster^24^) followed by quantification using inductively coupled plasma emission spectrometry (Instruments by wavelength range 167 to 852 nm, resolution 7 pm, high-performance solid-state CID821 detector; Thermo Scientific™ iCAP™ PRO Series ICP-OES, Thermo Fisher Scientific, Inc.). The cyanide in FC was determined using spectrophotometry (Instruments by wavelength range 330–1000 nm, spectral bandwidth 6 nm, wave length accuracy ± 1 nm, stray light <  − 0.5% T at 340 nm and 400 nm, silicon photodiode detector; Spectro SC, LaboMed, Inc.) with 2, 4-quinolinediol-pyridine reagent^25^.

Ruminal fluid samples of approximately 180 ml were pumped out using a modified vacuum via a stomach tube. The collection of ruminal fluid samples was carried out at two time sets (0 h pre-feeding and 4 h after morning feeding). Ruminal pH was assessed immediately (Hanna pH meter, HI-8424, Woonsocket, USA). The ruminal fluid samples were separated into three parts for the ruminal fermentation study. The first part of the samples was kept in a plastic bottle consisting of one molar H_2_SO_4_ at a 1:5 ratio and centrifuged for 15 min at 4 °C at a speed of 1600*g* to analyze the VFA and NH_3_-N concentration. After centrifugation, the upper and clear sample solution was collected and frozen at − 20 °C until measurement. Analysis of NH_3_-N (ID 991.20) concentration followed the method of AOAC^22^ as described in a previous study by Prachumchai et al.^10^. The VFA concentration was measured following a modified method of Osaki et al.^26^ using high-performance liquid chromatography as detailed in the procedure by So et al.^27^. The second part of the ruminal fluid sample was kept in plastic bottles consisting of 10% formalin at a 1:9 ratio to study protozoa and bacterial populations using a hemocytometer (Boeco, Hamburg, Germany)^28^. The third part of the ruminal fluid sample was kept at 4 °C to culture cyanide-utilizing bacteria^16^.

On the last day of each period, 10 ml were withdrawn from the jugular vein at 0 h pre-feeding and 4 h after the morning feeding and divided into two parts: the first 5 ml blood portion was kept into EDTA tubes and stored in refrigerator at a temp of 1–6 °C until BUN analysis, according to Crocker^29^, and another part of 5 ml blood was kept in tubes and centrifuged immediately to separate serum used later for blood thiocyanate analyzed using the method of Lambert et al.^30^. The blood samples were processed by centrifugation for 15 min at 500*g* prior to analysis.

### Cyanide-utilizing bacteria culture

Cyanide-utilizing bacteria were cultured according to Prachumchai et al.^10^. The nutrient medium was prepared according to Kandasamy et al.^31^ and Moradkhani et al.^32^. Briefly, 10 ml of ruminal fluid samples from each animal was diluted with 0.85% sodium chloride to 1:10, 1:100, and 1:1000 and pipetted, spread on agar plates, and incubated at 39 °C for 96 h. Then, cyanide-utilizing bacteria were counted according to the method of Kozaki et al.^33^ and expressed as cfu/g fresh matter (FM).

### Milk sampling, milk composition, and milk thiocyanate

Milk samples were collected during the last 7 day of each period to analyze milk composition and thiocyanate residue. The milk samples were obtained in a volume of 100 ml by mixing samples from morning-milking and afternoon-milking at a 60:40 ratio and stored in plastic bottles consisting of potassium dichromate at 0.2 g 100 mL^−1^ milk sample and stored at 4 °C. Milko-Scan33 (Foss Electric, Hillerod, Denmark) was used to analyze milk compositions, and Fossomatic 5000 Basic was used to analyze the somatic cell count. The data for protein, lactose, fat, solid not fat, total solids, and somatic cell count were provided. Milk urea nitrogen was determined using automated infrared analysis (Milcoscan, FOSS Electric A/S, Denmark) according to McCullough^34^. The milk thiocyanate concentration was measured according to Jacob et al.^35^.

### Statistics

Data of intake, digestibility, milk yield, and milk quality were analyzed using the *Proc Mixed* procedure of SAS^36^ as shown in the model:

$${\text{Y}}_{{{\text{ijkl}}}} = \upmu \, + {\text{ S}}_{{\text{l}}} + {\text{ P}}_{{\text{i}}} \left( {{\text{S}}_{{\text{l}}} } \right) \, + {\text{ Gk}}\left( {{\text{S}}_{{\text{l}}} } \right) \, + {\text{ T}}_{{\text{j}}} + \, \varepsilon_{{{\text{ijkl}}}}$$

where Y_ijkl_ is each observation for a given variable, μ is the overall mean, S_l_ is the random effect of square l; P_i_(S_l_) is the random effect of period i within square l; G_k_(S_l_) is the random effect of cow k within square l; T_j_ is the fixed effect of treatment and ε_ijkl_ is the residual random term.

Data of rumen fermentation characteristics and blood metabolites were analyzed using the *Proc Mixed* procedure of SAS^36^ as shown in the model:

$${\text{Y}}_{{{\text{ijklm}}}} = \upmu \, + {\text{ S}}_{{\text{l}}} + {\text{ P}}_{{\text{i}}} \left( {{\text{S}}_{{\text{l}}} } \right) \, + {\text{ Gk}}\left( {{\text{S}}_{{\text{l}}} } \right) \, + {\text{ t}}_{{\text{m}}} + {\text{ T}}_{{\text{j}}} + \, \varepsilon_{{{\text{ijkl}}}}$$

where Y_ijkl_ is each observation for a given variable, μ is the overall mean, S_l_ is the random effect of square l; P_i_(S_l_) is the random effect of period i within square l; G_k_(S_l_) is the random effect of cow k within square l; t_m_ is the random effect of sampling time at 0 h pre- and 4 h post-feeding T_j_ is the fixed effect of treatment and ε_ijkl_ is the residual random term.

Orthogonal polynomial were used to study the trend effect of FC replacement levels.
